# A closer look to neural pathways and psychopharmacology of obsessive compulsive disorder

**DOI:** 10.3389/fnbeh.2023.1282246

**Published:** 2023-11-16

**Authors:** Steven P. Gargano, Melody G. Santos, Sydney M. Taylor, Irene Pastis

**Affiliations:** ^1^East Carolina University Brody School of Medicine, Greenville, NC, United States; ^2^Internal Medicine and Psychiatry Combined Program, Department of Psychiatry and Behavioral Medicine, East Carolina University, Greenville, NC, United States; ^3^East Carolina University Brody School of Medicine, Greenville, NC, United States; ^4^Department of Psychiatry and Behavioral Medicine, East Carolina University, Greenville, NC, United States

**Keywords:** neural pathways, OCD, psychopharmacology, compulsion, models of compulsive behavior

## Abstract

The intricate neural pathways involved in obsessive-compulsive disorder (OCD) affect areas of our brain that control executive functioning, organization, and planning. OCD is a chronic condition that can be debilitating, afflicting millions of people worldwide. The lifetime prevalence of OCD in the US is 2.3%. OCD is predominantly characterized by obsessions consisting of intrusive and unwanted thoughts, often with impulses that are strongly associated with anxiety. Compulsions with OCD encompass repetitive behaviors or mental acts to satisfy their afflicted obsessions or impulses. While these factors can be unique to each individual, it has been widely established that the etiology of OCD is complex as it relates to neuronal pathways, psychopharmacology, and brain chemistry involved and warrants further exploration.

## Introduction

The intricate neural pathways involved in obsessive-compulsive disorder (OCD) affect areas of our brain that control executive functioning, organization, and planning. OCD is a chronic condition that can be debilitating, afflicting millions of people worldwide. The lifetime prevalence of OCD in the US is 2.3% (Obsessive-Compulsive Disorder (OCD), 2017). OCD is predominantly characterized by obsessions consisting of intrusive and unwanted thoughts, often with impulses that are strongly associated with anxiety. Compulsions with OCD encompass repetitive behaviors or mental acts to satisfy their afflicted obsessions or impulses (Stein et al., 2019). While these factors can be unique to each individual, it has been widely established that the etiology of OCD is complex as it relates to neuronal pathways, psychopharmacology, and brain chemistry involved and warrants further exploration.

### Areas of the brain implicated in OCD

With the use of imaging modalities such as positron emission tomography (PET), single-photon emission computerized tomography (SPECT), and functional magnetic resonance imaging (fMRI), we have elucidated some of the areas of the brain involved in OCD. The areas involved are the orbitofrontal cortex (OFC), anterior cingulate cortex, caudate nucleus, and thalamus (Saxena et al., 2001; Maia et al., 2008), with these structures connected via established neuroanatomic circuitry (Alexander et al., 1986). Recent findings from animal studies also indicate that other areas, such as the hypothalamus (Mangieri et al., 2018; Cassidy et al., 2019; Islam et al., 2022), hippocampus (Thompson et al., 2019; Mu et al., 2020), amygdala (Hong et al., 2014; Ullrich et al., 2018; Sun et al., 2019; Folkes et al., 2020), and spinal cord are involved as well (Xie et al., 2022).

Saxena et al. reviewed early neuroimaging studies in patients with OCD that were completed during rest, symptom provocation, as well as pretreatment and post-treatment (Saxena and Rauch, 2000). Baseline PET studies included in their review have uncovered significantly increased metabolic activity in the bilateral OFC (Baxter et al., 1987, 1988; Nordahl et al., 1989; Swedo et al., 1989; Sawle et al., 1991), basal ganglia (Baxter et al., 1987, 1988; Perani et al., 1995), and thalamus (Swedo et al., 1989; Perani et al., 1995), with comorbidity of significant depression potentially altering results (Baxter et al., 1987, 1988; Baxter et al., 1990; Martinot et al., 1990). The baseline SPECT studies reveal abnormalities in the frontal cortex (Machlin et al., 1991; Rubin et al., 1992; Lucey et al., 1997) and basal ganglia, particularly the caudate (Rubin et al., 1992; Adams et al., 1993; Lucey et al., 1997), while MR Spectroscopy found abnormal activity in the anterior cingulate cortex (Ebert et al., 1997) and striatum (Ebert et al., 1997; Bartha et al., 1998). For the neuroimaging symptom provocation studies, there is a robust positive correlation between OCD symptomatology and activation of the OFC, while the findings involving the basal ganglia, thalamus, limbic and paralimbic structures are less concordant (Zohar et al., 1989; McGuire et al., 1994; Rauch et al., 1994; Hollander et al., 1995; Breiter et al., 1996; Cottraux et al., 1996). Lastly, neuroimaging studies on pretreatment and post-treatment patients have shown that, regardless of treatment modality, there is decreased activity in both the OFC (Hollander et al., 1989; Benkelfat et al., 1990; Baxter et al., 1992; Squire, 1992; Schwartz et al., 1996; Saxena et al., 1999) and caudate post-treatment (Hollander et al., 1989; Benkelfat et al., 1990; Baxter et al., 1992; Schwartz et al., 1996; Moore et al., 1998; Saxena et al., 1999).

In a review of the role of dopamine in OCD, Koo et al. examined neuroimaging studies in the context of OCD pathophysiology (Koo et al., 2010). Volumetry reports have determined that the OFC, globus pallidus, anterior cingulate cortex (ACC), caudate, and thalamus were decreased in volume, with these areas consisting of the frontostriatal circuit (Scarone et al., 1992; Robinson et al., 1995; Szeszko et al., 1999; Kwon et al., 2003; Choi et al., 2004; Kang et al., 2004). SPECT/PET research has shown increased metabolic rates in the mediofrontal cortex in OCD, specifically the dorsal parietal cortex, left posterofrontal cortex, OFC, left inferofrontal cortex, medial frontal cortex, and left parietal cortex (Rubin et al., 1992; Harris et al., 1994; Lucey et al., 1995). These findings suggest the frontostriatal circuit, along with the basal ganglia, is the primary brain region altered in OCD pathology following treatment (O’Regan, 1970; Rivers-Bulkeley and Hollender, 1982; Swedo et al., 1992).

Additional PET studies on treated and treatment-naive patients found similar metabolic rates in the OFC and caudate, areas closely linked to reward and learning processing and rich in dopaminergic and serotonergic nerve fibers (Benkelfat et al., 1990; Hansen et al., 2002). Furthermore, dopamine transporter binding abnormalities in OCD patients have been found in the putamen and caudate, while dopamine transporter availability abnormalities were found to be replicated in the striatum of treatment-naive OCD patients (van der Wee et al., 2004; Hesse et al., 2005; Kim et al., 2007). Following SSRI treatment in particular, dopamine transporter expression is found to be increased, indicating that pharmacological agents in OCD treatment act through the dopaminergic system and that there is a reciprocal action of dopamine and serotonin in the subcortex of patients with OCD (Pogarell et al., 2005; Kim et al., 2007).

It is important to note that there appears to be varying involvement of the subregions of the OFC and ACC in the pathophysiology of OCD (Milad and Rauch, 2012). Studies utilizing fMRI have found hyperactivity of the lateral OFC (LOFC) to be positively correlated with symptom severity in OCD subjects, with the medial OFC (mOFC) appearing inversely correlated (Adler et al., 2000; Milad and Rauch, 2007; Rauch et al., 2007). The lesser extent of hyperactivity in the LOFC prior to selective serotonin reuptake inhibitor (SSRI) treatment has also been associated with better treatment response (Rauch et al., 2002). Although other fMRI studies appear to contradict this model by showing hypoactivation in the LOFC (Remijnse et al., 2006; Chamberlain et al., 2008), they all still suggest that there is dysfunction in both the lateral and medial regions of the OFC in OCD. The dorsal region of the ACC (dACC) is the region of the ACC found to be most relevant to the psychopathology of OCD (Milad and Rauch, 2012). Studies support hyperactivity of the dACC in OCD (Fitzgerald et al., 2005, 2010; Schlösser et al., 2010), and SSRI treatment-responsive OCD patients have been found to have reduced metabolism in the dACC following treatment (Perani et al., 1995).

In a recent study analyzing the resting-state functional connectome of OCD patients, Bruin et al. further strengthen findings of regional involvement in the previous studies using machine learning (Graybiel and Rauch, 2000; Milad and Rauch, 2012; van den Heuvel et al., 2016; Bruin et al., 2023). However, their results show a lesser degree of subcortical involvement in OCD and suggest the most significant hypo-connectivity to be found within the cortico-striato-thalamo-cortical (CSTC) sensorimotor network when measured via resting-state fMRI (Bruin et al., 2023). With these new innovative models incorporating machine learning with fMRI to map the involvement of brain areas in OCD patients, new pathways are open in the field of OCD research to locate key regions and identify specific circuitry to target in the development of novel pharmacological treatments and show potential to provide a stronger understanding of the pathophysiology behind OCD.

Animal studies are opening the doors to previously unexplored areas of involvement in OCD through the study of OCD-like behaviors. Hypothalamic involvement is one region that has been proposed through this research, particularly in the lateral hypothalamus (LH) and paraventricular hypothalamus (PVH) (Mangieri et al., 2018; Cassidy et al., 2019; Islam et al., 2022). Additionally, the ventral subiculum of the hippocampus (Thompson et al., 2019; Mu et al., 2020), the posteromedial and basolateral subdivisions of the amygdala (Hong et al., 2014; Ullrich et al., 2018; Sun et al., 2019; Folkes et al., 2020), and the trigeminal nucleus of the spinal cord (Xie et al., 2022) have all gained support through animal studies in having regional involvement in the psychopathology of OCD. More will be discussed on the specific circuitry uncovered through these studies in the *Pathways* section below.

### Pathways

The most widely accepted albeit intricate pathways that have thus far been elucidated involve a CSTC loop as the core mechanism in the pathophysiology of OCD (Alexander et al., 1986; Middleton and Strick, 2001; Milad and Rauch, 2012; Goodman et al., 2021). The current evidence suggests that OCD is a disorder where its dysfunction is a byproduct of defective neural networks rather than a single region of the brain (Goodman et al., 2021). Obsessions and compulsions can vary widely in terms of the nature of the thoughts, mental acts, motives, and drives behind the obsessive thought that then provokes/leads to the compulsive behavior or mental act. Based on the nature and the presentation of obsessions and compulsions it is reasonable to expect involvement of different neuronal pathways.

Milad and Rauch have established a conceptual and investigatory framework for understanding these networks and analyzing their dysfunctions in OCD using the CSTC loops motif and associated brain regions (Milad and Rauch, 2012). Their proposed framework consists of 3 circuit loops: affective circuit (ACC/ventromedial PFC, nucleus accumbens, thalamus), involved in affective and reward processing; dorsal cognitive circuit (dorsolateral PFC, dorsal caudate, thalamus), involved in working memory an executive function; ventral cognitive circuit (anterolateral OFC, putamen, thalamus), involved in motor response and inhibition. The use of this framework has largely shown consistent results with the imaging studies reviewed in the previous section (Milad and Rauch, 2012). Other conceptual frameworks have been proposed such as the more recent neurocircuit-based taxonomy proposed by Shephard et al. for guidance in the treatment of OCD (Shephard et al., 2021). This model consists of the following 5 circuits and their associated functions: fronto-limbic (amygdala and ventromedial PFC), emotional responses such as fear and anxiety; sensorimotor (supplementary motor area, putamen, thalamus), motor behavior and sensory integration; ventral cognitive (inferior frontal gyrus, ventrolateral PFC, ventral caudate, thalamus), control of self-regulatory behavior; ventral affective (orbitofrontal cortex, nucleus accumbens, thalamus), reward processing and response; dorsal cognitive (dorsolateral PFC, dorsomedial PFC, dorsal caudate, thalamus), executive function and emotional regulation. These frameworks can serve as guides in better understanding the complex interplay of these networks in OCD when conducting imaging, animal, and clinical research.

As stated in the previous section, animal models of OCD-like behavior play an important role in uncovering potential brain regions and specific pathways, their function, and neurotransmitters associated with OCD neuropsychopathology. In a recent animal study examining midbrain dopaminergic neurons and OCD-like behavior, Xue et al. identified the location and circuitry of repetitive behaviors in mice OCD animal models (Xue et al., 2022). The results indicate dopaminergic neuronal projections from the substantia nigra pars compacta (SNc) to the ventromedial striatum (VMS) and LOFC control repetitive behavior via a dual-gating mechanism. Grooming behavior in mice is modulated by CSTC circuit dysfunction and is reasoned to be a stereotypical behavior involved in OCD (Robinson et al., 1995; Kang et al., 2004; Szeszko et al., 2004). Xue et al. found that the dopaminergic projections of the SNc-VMS pathway act on D1 receptors to promote grooming, while projections of the SNc-LOFC pathway act on D2 receptors to suppress grooming, uncovering this reciprocal acting dual-gating function. While approximately half of OCD patients fail to have a clinically significant response to SSRI treatment in practice (Jenike, 2004), these treatment-resistant patients have displayed a response to dopamine antagonists (Maina et al., 2008; Goodwin et al., 2009). Although it is unknown whether dopaminergic alteration is ubiquitous among patients, these findings suggest potential novel target regions for future pharmacologic and brain stimulation interventions in the treatment of OCD.

In a study examining hypothalamic involvement in compulsive behavior in transgenic mice, Mangier et al. uncovered a dual circuit originating from the lateral hypothalamus (LH) and targeting the paraventricular hypothalamus (PVH) that modulates feeding and compulsive self-grooming (Mangieri et al., 2018). They found GABAergic LH → PVH stimulation to promote feeding while glutamatergic stimulation induces self-grooming, with rapid shifts from stress-induced self-grooming in GABAergic activation and from fasting-induced feeding in glutaminergic activation, suggesting a shared neural pathway underpinning both behaviors and implicating LH-PVH connections in these compulsive actions (Mangieri et al., 2018). Additional animal studies have shown further support for the involvement of both the LH (Cassidy et al., 2019) and PVH (Islam et al., 2022) in these behaviors.

The hippocampus has also been an area of interest in recent animal studies with a newly discovered di-synaptic circuit linking the hippocampal ventral subiculum to the ventral lateral septum and then the hypothalamus tuberal nucleus, found to be involved in regulating stress-induced self-grooming behaviors (Mu et al., 2020). Additionally, in a study on OCD-related behaviors and the BTB/POZ domain-containing 3 (BTBD3) transcription factor, a potential risk gene for OCD and highly expressed in limbic CSTC circuits, it has been found that hippocampal BTBD3 expression selectively modulates both compulsive-like and exploratory behavior in mice (Thompson et al., 2019). BTBD3 has been found to guide dendrites toward active axon terminals and regulates the activity-dependent pruning of dendrites in the primary sensory cortex during neonatal development, aiding in the formation of neural circuitry (Matsui et al., 2013). However, it remains uncertain whether BTBD3 plays a similar role in other brain regions, particularly the limbic CSTC circuits (Thompson et al., 2019).

The role of the amygdala in OCD has also gained more attention with animal studies showing glutamatergic activity in the posterior subdivision of the medial amygdala influencing self-grooming behaviors (Hong et al., 2014), potential thalamo-amygdala circuitry involvement in self-grooming behaviors (Ullrich et al., 2018), and projections from the basolateral amygdala to both the medial prefrontal cortex and nucleus accumbens influencing checking behaviors and self-grooming behaviors, respectively (Sun et al., 2019; Folkes et al., 2020). Lastly, although the involvement of the spinal cord in these behaviors was previously largely unknown, new findings released by Xie et al. uncover a neural circuit from the caudal aspect of the spinal trigeminal nucleus to the cervical spinal cord found to maintain repetitive self-grooming behaviors in mice (Xie et al., 2022). Although the extent of proposed regional neural involvement in OCD-like behaviors supports the multifaceted complexity of the disorder, further research is needed to identify the implications of these pathways in human subjects.

### Cell types and molecules (neurotransmitters)

In patients with OCD, their symptoms may be attributed to neurotransmitters including serotonin and dopamine predominantly, as evidenced in multiple studies. Serotonin 1B and 1D receptors are implicated in the exacerbation of OCD symptom severity. This is well-established, as SSRIs are the first-line therapy in patients with OCD and are shown to benefit nearly 50% of patients and improve their outcomes (Soomro et al., 2008; Pittenger et al., 2011; Okutucu et al., 2023). Furthermore, when serotonin 1B and 1D receptors are stimulated, OCD symptoms are profoundly exacerbated (Koran et al., 2001; Gross-Isseroff et al., 2004; Zohar et al., 2004; Pittenger et al., 2011).

Studies have demonstrated that dopamine and dopaminergic systems in the midbrain play a key role in OCD; specifically, patients experience an activation of D1 receptors. Studies in rodents have found a decrease in excessively stereotyped grooming or “OCD-like” activities when D1 receptors have been knocked out (Berridge and Aldridge, 2000; Zike et al., 2017; Xue et al., 2022). Additionally, this has been determined by receptor binding research using radioisotopes; evidence indicates that dopamine transporter binding ability is compromised in OCD, specifically in subcortical areas of the putamen and caudate (van der Wee et al., 2004; Koo et al., 2010). Overall, studies have shown that monotherapy with SSRIs in treating OCD may not fully treat patients, and many patients benefit from the addition of an antipsychotic medication that displays a dopaminergic mechanism of action due to the interaction of serotonin and dopamine (Korsgaard et al., 1985; Koo et al., 2010).

Recent clinical trials have aimed at targeting the glutaminergic system (O’Neill, 2020). Glutamate is the primary excitatory neurotransmitter in the brain of adults and its dysfunction has been identified as a potential link to the etiology of OCD (Pittenger et al., 2011). In addition to the inhibitory neurotransmitter GABA, glutamatergic pathways play a crucial role in the intricate connections within the CSTC circuit, as stated previously to be implicated in the development of OCD (Goodman et al., 2021). In addition, several glutamate-related genes have been associated with OCD risk and have been studied for decades (Pittenger et al., 2011).

### Treatment

There are several published guidelines for the management of OCD. These include the American Psychiatric Association, Canadian Psychiatric Association, and National Institute for Health and Clinical Excellence (National Institute for Health and Care Excellence, 2005; Koran et al., 2007; Katzman et al., 2014). Pharmacotherapy with SSRIs and cognitive-behavioral therapy are considered the standard first-line treatments for OCD.

Glutamate-modulating pharmacotherapy such as U.S. FDA-approved Riluzole, for example, was one of the first of these agents to be tested in the treatment of OCD, with the overall effect exhibiting increased glutamate clearance (Goodman) (Goodman et al., 2021). In addition, studies have suggested that this treatment for OCD may be efficacious for many common comorbid conditions, including major depressive disorder, bipolar depression, and generalized anxiety (Pittenger et al., 2011).

### Pharmacotherapy

A meta-analysis comparing the effectiveness of SSRIs and placebo showed that SSRIs are effective for treating OCD (Soomro et al., 2008). Skapinakis et al. completed a network meta-analysis and determined there were no significant differences between SSRIs in the treatment of OCD (Skapinakis et al., 2016). Usually, higher doses of SSRI are needed for OCD as compared to depression (Bloch et al., 2010). If the patient is unresponsive to the first SSRI, guidelines recommend a trial of a second SSRI. Most recommendations advise switching to a second-line choice if a second SSRI fails. These include venlafaxine or clomipramine. Although previously considered to be a first-line agent, clomipramine is a second-line agent due to its more difficult side effect profile. In the US and Canada, mirtazapine is also recommended as a second-line alternative medication (Koran et al., 2005; van Roessel et al., 2023). A meta-analysis in 2015 found augmentation of SSRIs with antipsychotics to be beneficial, particularly with haloperidol, aripiprazole, and risperidone (Dold et al., 2015). A literature review suggested there may be some benefit to using ondansetron as shown in five therapeutic studies (Serata et al., 2015).

Memantine is being considered for the treatment of OCD although not yet established. In a systematic review, Modarresi et al. concluded that augmentation with memantine was safe and effective for OCD treatment in moderate to severe disease (Modarresi et al., 2019). Riluzone and ketamine are also being considered for use in refractory OCD (Pittenger, 2021). Sharma et al. used several IV ketamine infusions to treat SSRI-resistant OCD. They found that the Yale-Brown Obsessive Compulsive Scale (YBOCS) total score significantly decreased. Control trials with larger sample sizes are required to investigate the effectiveness of ketamine and find indicators of ketamine responsiveness (Sharma et al., 2020). Before any recommendations for the use can be made for these medications, more comprehensive, higher-quality investigations are required.

### Non-invasive brain stimulation

Despite the effectiveness of current pharmacological and behavioral treatments, many patients are still unresponsive. Steuber et al. set out to evaluate the benefit of repetitive transcranial magnetic stimulation (rTMS) on OCD patients. The study showed that when compared to sham, rTMS showed a 3 times greater probability of treatment response and a modest therapeutic impact for the severity of OCD symptoms. The therapeutic effects of rTMS on the intensity of OCD symptoms were correlated with improvements in comorbid depression severity. To maximize treatment, it is crucial to take into account the variables that affect the therapeutic benefits (Steuber and McGuire, 2023).

A total of two hundred and nineteen OCD patients participated in a post-marketing investigation to assess the effectiveness in real-world practice. The response measured as at least a 30% decrease in Y-BOCS score from baseline to the endpoint, was the main outcome. First response and sustained response for 1 month. After 29 sessions, 22 clinical sites discovered a response rate of 72.6%. Continued reduction of OCD symptoms was seen by prolonging the course past 29 sessions. Hence, showing the possibility of usefulness for treatment in non-responders. In this study, physicians had the possibility of utilizing augmentation medications or increasing the rate of therapy which has not been permitted in sham-controlled studies. This may have been a contributing factor in the increased response rate observed in real-world practice (Roth et al., 2021).

### Psychotherapy

The only type of psychotherapy for which there is solid evidence in OCD is cognitive-behavioral therapy, which is also the most effective treatment for OCD. This is most likely because the most effective form of treatment for OCD, behavioral therapy is a cornerstone of cognitive-behavioral therapy. The most crucial element of CBT is exposure response therapy. Hence, guidelines recommend using CBT as a first-line treatment option.

### Models of compulsive behavior

OCD has long been associated with a plethora of compulsions that are central to symptomatology and disease subtypes. Compulsive behavior and obsessional thoughts are not only associated with OCD, but are present in several other neuropsychiatric disorders sometimes referred to as “impulsive-compulsive disorders,” including but not limited to: OCD, Tourette syndrome, addiction, substance use disorders, behavioral addiction such as gambling and internet addiction, and compulsive eating (Robbins et al., 2019). Models of different compulsions are being studied, including but not limited to foraging, hoarding, grooming, washing, checking, drug seeking, feeding, mating-related, aggression, gaming, and smartphone use (Radomsky et al., 2007; Figee et al., 2016; Robbins et al., 2019; Benaroya-Milshtein et al., 2020; Kuty-Pachecka, 2021).

Animal models, including genetic, pharmacological, ethological, and stress-induced models, are frequently used to study compulsive behaviors and assist in the development of our current understanding of the neuropsychological basis of OCD and compulsive behaviors (Camilla d’Angelo et al., 2014; Robbins et al., 2019). d’Angelo et al. identified some of these mentioned animal model subtypes in their review on OCD. Ethological models provide researchers with the ability to study naturally occurring behavioral processes compared to those that are artificially induced (Camilla d’Angelo et al., 2014), such as canine acral lick dermatitis in dogs which is a representative model of excessive grooming behavior (Rapoport et al., 1992). Drug-induced behavioral models can be used to induce specific OCD symptoms in humans such as compulsive checking, increased anxiety, indecision, and preservation (Camilla d’Angelo et al., 2014). An example of this can be seen in rats treated with sub-chronic quinpirole, a D2/D3 receptor agonist, which induces increased checking behavior (Szechtman et al., 1998; Eagle et al., 2014). Genetic models are more difficult to use due to the presumed polygenetic involvement and varying heritability estimates in OCD (Jonnal et al., 2000; Hettema et al., 2001; Camilla d’Angelo et al., 2014). Knockout of the Sapap3 gene in mice is one of the most studied genetic models used in OCD research and induces excessive grooming and anxiety (Burguière et al., 2013; Pinhal et al., 2018). In a recent study, Manning et al. found a correlation between deficits in reversal learning and increased c-fos activity within the medial PFC of this model, associating it with the correlation between deficits in fear reversal increased vmPFC activity in OCD patients (Apergis-Schoute et al., 2017; Manning et al., 2019). Although no single model can be used as an all-inclusive representative of OCD, the different models allow for the study of specific symptomatology and are useful in acquiring a better understanding of pathophysiology for individual subsets as well as assisting with the development of targeted pharmacologic interventions.

In a network analysis of obsessive-compulsive symptoms and beliefs, using the OBQ-44 and OCI-R to uncover those most central to OCD, Bunmi et al. found that “*having intrusive thoughts means I’m out of control”* and “*having nasty thoughts means I am a terrible person”* to be the most central and statistically significant symptoms within the network (Olatunji et al., 2019). These findings support past research suggesting that distorted beliefs surrounding an individual’s thoughts is more predictive of OCD symptoms, rather than dysfunctional beliefs concerning perfectionism or uncertainty (Myers et al., 2008). Although these core symptoms are representative of distorted beliefs, these obsessive beliefs are thought to contribute to and predict the development of OCD symptoms over time (Rachman, 1998; Salkovskis, 1998; Abramowitz et al., 2006). Intriguingly, hoarding, although traditionally identified as a symptom of OCD, was found to have low centrality in the network and empirical studies do not show consistency in its relationship with OCD (Grisham et al., 2005; Wu and Watson, 2005; Saxena, 2007; Abramowitz et al., 2008).

Sexual obsessions in OCD are characterized by egodystonic intrusive thoughts or images that can include sexual content related to inappropriate sexual activity with family, children, or animals, fears surrounding sexual orientation, or aggressive sexual behaviors (Williams, 2008; Real et al., 2013). It is important to note that these obsessions are not pleasant for the patient, and the associated compulsions do not bring pleasure, but instead reduce anxiety (Kuty-Pachecka, 2021). The patient finds themself acting out the compulsion in an attempt to gain control over the activity of their mind and to neutralize the negative emotions that arise as a consequence of feeling responsible for the obsession, which, as a result, increases the probability of future intrusions and consolidates the belief of responsibility (Salkovskis, 1999). In a study examining trait anger and anger expression in individuals with OCD with primary checking compulsions, Radomsky et al. found that trait anger, but not anger expression, was greater in these individuals compared to controls (Radomsky et al., 2007). Studies have also found that children with tic disorders and comorbid OCD have an increased probability of aggressive behavior compared to those with tic disorder alone (Budman et al., 2000; Freeman et al., 2000; Budman et al., 2015; Benaroya-Milshtein et al., 2020).

OCD has also been proposed by some researchers to be conceptualized as a behavioral addiction (Holden, 2001; Denys et al., 2004), as well as other disorders that share compulsivities such as pathological gambling, compulsive eating, sexual behavior, and computer use (Holden, 2001; Grant et al., 2006). In a review on compulsivity in OCD and addictions, Figee et al. examined the neurobiologic overlap between compulsivity in OCD, substance-use disorders, and behavioral addictions, as stated above (Figee et al., 2016). Their reviewed data suggests that compulsivity in these disorders involves dysfunctional reward and punishment in the ventral striatum with associated attenuation of dopamine release (Figee et al., 2011, 2013), along with negative reinforcement within the limbic system (Kennett et al., 2013; Koob, 2015), providing a potential explanation for the involvement of repetitive self-destructive behaviors (Figee et al., 2016). This compulsivity is also found to involve cognitive and behavioral inflexibility (Chamberlain et al., 2006; Menzies et al., 2007), with the possible underlying mechanism of co-occurring impairment of top-down regulation in the vmPFC (Figee et al., 2013; Harrison et al., 2013; Sakai et al., 2020), serotonergic defects in the prefrontal cortex (Figee et al., 2010; Pelloux et al., 2012), as well as excessive dopamine and glutamate signaling (Wu et al., 2012; Sesia et al., 2013). Lastly, they show that habitual responding plays a role in compulsivity with imbalances between ventral and dorsal frontostriatal recruitment (Everitt and Robbins, 2005; Everitt et al., 2008; Gillan et al., 2011, 2014; Willuhn et al., 2012; Sjoerds et al., 2013; Voon et al., 2015).

### Anxiety and compulsive behavior

Anxiety and anxiety disorders, including generalized anxiety disorder (GAD), are strongly associated with OCD not only epidemiologically, but also within clinical settings (Fontenelle and Hasler, 2008; Sharma et al., 2021). Anxiety and compulsive behaviors as seen in conditions like OCD have many similarities and most predominantly overlap in the notion of repetitive and intrusive thoughts; chronic worrisome thoughts in GAD are thought to be similar to unwanted obsessions in OCD (Sharma et al., 2021; American Psychiatric Association, 2022). In addition, the main function of compulsive behavior within OCD is often to relieve unwanted anxiety (Starcevic et al., 2011). Many mental processes within OCD begin in response to having anxiety and are centered around preventing it from occurring (American Psychiatric Association, 2022; Okutucu et al., 2023). However, distinctive features between anxiety and compulsive behaviors exist. In those who have anxiety, worries commonly involve rational and logical thoughts occurring in one’s daily life and the future. Conversely, intrusive thoughts and compulsive behaviors within OCD are ego-dystonic and may be bizarre (Lee and Kwon, 2003; Sharma et al., 2021).

### Movement disorders and OCD

Relationships between movement disorders and OCD have been widely established, as many of the same neurotransmitters are implicated in the pathophysiology of both and exhibit great overlap. In patients with idiopathic Parkinsonism, when given medications such as L-dopa, OCD symptoms can be exhibited and aggravated due to its action on the basal ganglia (Andén et al., 1970; Sacks and Kohl, 1970; Koo et al., 2010). Tourette’s syndrome also has its origin in the basal ganglia and frontal cortex. In children with Tourette’s, over half also have remarkable OCD symptoms and meet diagnostic criteria. Additionally, obsessive-compulsive symptoms were found to be more rampant in Huntington’s disease, independent of movement disorder manifestations (Beglinger et al., 2007; Fibbe et al., 2012). This neurotransmitter overlap is not limited to movement disorders, as many medical diseases may also present with OCD-like features when affecting the basal ganglia, including Sydenham’s chorea from rheumatic fever secondary to group A beta-hemolytic streptococcal infection.

### Conclusion and clinical trials

In this review we summarized implicated areas of the brain, potential novel target regions for future pharmacologic and brain stimulation intervention, pathways and neurotransmitters involved, and models of compulsive behaviors in OCD. These neural pathways, psychopharmacology, and brain chemistry involved in the etiology of OCD are complicated and call for additional studies. Several clinical trials are currently under investigation. Reinhart and his team are conducting a clinical trial utilizing a novel neuromodulation method, based on reward-related rhythms of the OFC, for the reduction of OCD symptoms (Reinhart, 2023). Reinhart and his team are using high-definition transcranial alternating current stimulation (HD-tACS), a non-invasive brain stimulation technique that uses alternating current to modulate brain activity, guided by electroencephalogram (EEG) brain wave recordings to test whether repetitive modulation of relevant rhythm activity in the OFC can result in rapid and sustainable symptom production (Reinhart, 2023). The OBSESS trial is another clinical trial that is recruiting OCD patients who meet established surgical criteria to implant permanent DBS leads and temporary stereo-EEG electrodes to investigate personalized DBS programming (Sheth, 2023). The trial seeks to demonstrate the efficacy of data-derived DBS programming in reducing symptoms while collecting chronic on-device recordings to understand physiological signatures, therapeutic response, uncover biomarkers reflecting symptom severity, and guide future therapies in OCD (Sheth, 2023). While there is still much to be uncovered in the field of OCD research, these trials shed light to the direction of novel interventions and provide hope to the millions who continue to suffer from OCD worldwide.

## Author contributions

SG: Writing – original draft, Writing – review & editing. MS: Conceptualization, Writing – original draft, Writing – review & editing. ST: Writing – original draft, Writing – review & editing. IP: Conceptualization, Supervision, Writing – original draft, Writing – review & editing.
